# NMR Studies of the Structure and Function of the HIV-1 5′-Leader

**DOI:** 10.3390/v8120338

**Published:** 2016-12-21

**Authors:** Sarah C. Keane, Michael F. Summers

**Affiliations:** Howard Hughes Medical Institute, Department of Chemistry and Biochemistry, University of Maryland Baltimore County, 1000 Hilltop Circle, Baltimore, MD 21250, USA; keanes@umbc.edu

**Keywords:** NMR, HIV-1, 5′-leader, RNA, genome, structure, function

## Abstract

The 5′-leader of the human immunodeficiency virus type 1 (HIV-1) genome plays several critical roles during viral replication, including differentially establishing mRNA versus genomic RNA (gRNA) fates. As observed for proteins, the function of the RNA is tightly regulated by its structure, and a common paradigm has been that genome function is temporally modulated by structural changes in the 5′-leader. Over the past 30 years, combinations of nucleotide reactivity mapping experiments with biochemistry, mutagenesis, and phylogenetic studies have provided clues regarding the secondary structures of stretches of residues within the leader that adopt functionally discrete domains. More recently, nuclear magnetic resonance (NMR) spectroscopy approaches have been developed that enable direct detection of intra- and inter-molecular interactions within the intact leader, providing detailed insights into the structural determinants and mechanisms that regulate HIV-1 genome packaging and function.

## 1. Dimerization-Dependent Control of Human Immunodeficiency Virus Type 1 (HIV-1) Genome Function

A hallmark of retroviruses is that they selectively and efficiently package two copies of their unspliced, 5′-capped and 3′-polyadenylated RNA genomes during virus assembly [1]. Both molecules are utilized for strand transfer-mediated recombination during reverse transcription [2,3], enabling transcription at strand breaks caused by restriction endonucleases [4] and promoting evolution of resistance to antiviral therapies [5]. Unspliced genomes are selected for packaging as non-covalently linked dimers from a cellular milieu that includes a substantial excess of non-viral and spliced viral mRNAs [6,7]. RNA elements that direct packaging are located within the 5′-leader of the genome [8], the most conserved region of the genome [9]. Elements important for transcriptional activation, splicing, and translation initiation are also located within the 5′-leader, and there is evidence that these and other activities are temporally modulated by dimerization-dependent exposure of functional signals [10,11,12,13,14].

**Figure 1:**
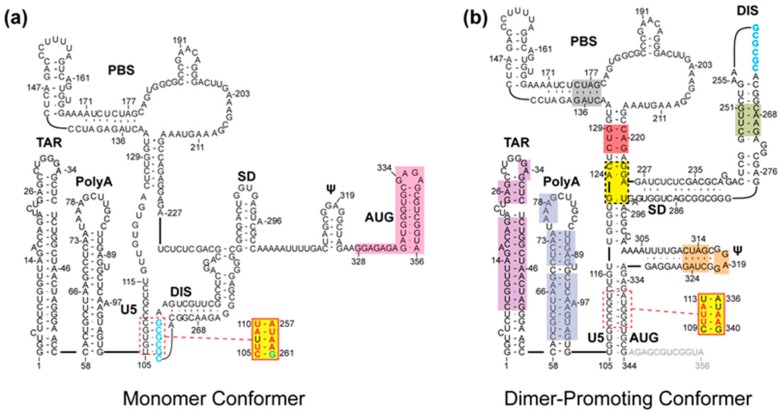
Secondary structure of the human immunodeficiency virus type 1 (HIV-1) 5′-leader in monomer (**a**) and dimer-promoting (**b**) conformations. Highlighted regions on each structure denote elements whose structure has been probed using nuclear magnetic resonance (NMR) spectroscopy in the full-length leader. In contrast to the dimeric form of the 5′-leader, few regions of the monomeric leader have been probed by NMR spectroscopy. Yellow boxes indicate regions whose structure could only be probed in truncated or mutated (see inset) 5′-leader constructs, and other colored boxes correspond to different regions with detectable Adenosine-H2 signals in partially deuterated 5′-leader RNA samples. Figure adapted from [31].

Dimerization occurs both in the cytoplasm and at the plasma membrane (PM), and is mediated by a conserved GC-rich palindromic dimer initiation sequence (DIS) located within the 5′-leader of the genome, Figure 1. Genetic studies indicate that the DIS is responsible for RNA:RNA partner selection [15,16], but other leader elements, including those overlapping the *gag* start codon (AUG, Figure 1), also play roles in dimerization [13,17,18,19]. Genome selection during virus assembly is mediated by interactions between the nucleocapsid (NC) domains of a small number of viral Gag polyproteins (~12 or fewer) [20] and packaging signals located within the 5′-leader of the viral RNA [8,10,11,14,21,22,23,24,25,26,27,28,29]. Additional Gag proteins associate with the genome after it is anchored to the PM, leading to further virus assembly and budding [30].

## 2. Probing the Secondary Structure of the Intact 5′-Leader by Nuclear Magnetic Resonance (NMR) Spectroscopy

In vitro, human immunodeficiency virus type 1 (HIV-1) 5′-leader RNA (residues 1–356, which includes the intact 5′-untranslated region and the first 21 nucleotides of the *gag* gene; Figure 1) exists as an equilibrium mixture of monomeric and dimeric species [8,10,11,14,21,22,23,24,25,26,27,28,29]. The secondary structure of the 5′-leader has been probed by combinations of nucleotide reactivity mapping, phylogenetic analyses, biochemical and molecular biological studies, and free energy calculations, resulting in more than 25 predicted secondary structures and multiple dimerization models [8]. Probing of the intact HIV-1 5′-leader RNA by nuclear magnetic resonance (NMR) spectroscopy was initially conducted with a segmentally labeled sample, in which residues overlapping the *gag* start codon (AUG; Figure 1) were enriched with ^13^Carbon (^13^C) [13]. These studies revealed that AUG adopts a hairpin structure in the monomeric form of the 5′-leader that is favored at low ionic strength [13]. Technical problems (severe line broadening associated with ^1^H-^13^C dipolar coupling) precluded ^13^C-based NMR spectroscopy studies of the dimeric form of the leader. However, using a ^2^H-edited approach that does not rely on ^13^C labeling (long-range probing by adenosine interaction detection, lrAID), residues of AUG were shown to base pair with an upstream U5 element in the dimeric form of the 5′-leader, consistent with phylogenetic predictions [32,33]. These data, in combination with other mutagenesis experiments, suggested a dimerization model in which DIS is sequestered by base pairing with U5 in the monomeric RNA, and U5:AUG base pairing displaces and exposes the DIS to promote dimerization (Figure 1) [13]. Mutations engineered to favor the monomer reduce the number of high affinity NC binding sites and inhibit vector RNA packaging, whereas those that promote dimerization also promote NC binding and packaging, consistent with the proposed dimerization dependent RNA structural switch mechanism. Subsequent studies with HIV-2 and simian immunodeficiency virus (SIV) 5′-leader RNAs indicate that this dimerization/packaging mechanism is likely conserved among evolutionarily distant lentiviruses [34].

## 3. NMR Structure of the HIV-1 RNA Packaging Signal

**Figure 2:**
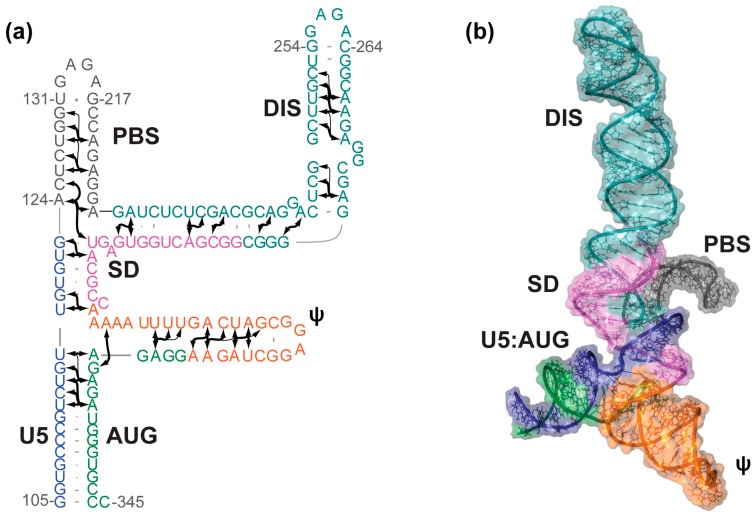
Solution structure of the HIV-1 Ψ^CESm^. (**a**) Secondary structure of the HIV-1 Core Encapsidation Signal (Ψ^CESm^) determined using a fragmentation-based ^2^H-edited approach. Arrows represent directly-detected nuclear Overhauser effects (NOEs) from adenosines to neighboring nuclei; (**b**) NMR-derived three-dimensional structure of Ψ^CESm^. The tandem three-way junction topology sequesters the major splice donor site and exposes the dimer initiation sequence (DIS). Figure adapted from [36].

A systematic nucleotide deletion strategy was used to identify a minimal region of the 5′-leader sufficient for packaging (Core Encapsidation Signal, Ψ^CES^) [35]. Ψ^CES^ lacks the TAR, PolyA, and the upper primer binding site (PBS) loops, yet maintains the dimerization, high affinity NC binding, and importantly, NMR spectroscopic properties observed for the intact, native 5′-leader [35]. Ψ^CES^ is also capable of directing vector RNA packaging with >80% efficiency of the native 5′-leader [35]. To improve NMR spectral quality for structural studies, the size of the symmetrical Ψ^CES^ dimer was effectively “halved” by substituting the native DIS palindromic loop by a hairpin-promoting tetraloop (Ψ^CESm^), Figure 2 [36]. This substitution did not affect the NC binding properties or nuclear Overhauser effect (NOE) NMR spectral patterns of the RNA. The structure of Ψ^CESm^ was determined using a fragmentation based ^2^H-edited NMR approach, which was designed to simplify spectral analysis and provide direct information about long-range base pairing interactions, Figure 2 [36]. Unexpectedly, Ψ^CESm^ was found to adopt a tandem three-way junction structure, in which residues of the splice donor site (SD) participate in long-range base pairing (a tandem three-way junction), rather than adopting a widely-predicted hairpin structure. Notably, the observed secondary structure is in better agreement with in-gel chemical probing results obtained for the resolved dimeric 5′-leader RNA [37] than with previously predicted secondary structures [38,39]. The Ψ^CESm^ structure informs four key aspects of HIV replication: (i) dimerization dependent attenuation of translation is explained by the sequestration of the *gag* start codon, which base pairs with upstream U5 residues; (ii) the tandem three-way junction exposes un-paired and weakly-paired guanosines that are required for high affinity NC binding, which explains why Ψ^CES^ promotes RNA packaging; (iii) the exquisite selectivity of HIV-1 to package its unspliced genome is likely due to the requirement of residues immediately downstream of the major splice site for formation of the packaging-competent three-way junction structure; and (iv) although the structure of the monomeric genome has not been determined, it is likely that monomers are ignored during virus assembly because they do not adopt the guanosine-exposing tandem three-way junction structure [36].

A phylogenetic analysis showed that 31 out of 48 residues at or near the tandem three-way junction are strictly (16 sites) or >99% (15 sites) conserved, and that 13 sites exhibited 90%–99% identity [36]. The lack of base pair covariation has led some to question the validity of the tandem three-way junction structure [40], especially in view of strong evidence that residues of SD adopt a hairpin structure [8,40,41]. Although it is unfortunate that sequence conservation was too high for a conclusive phylogenetic assessment, it is certainly possible (likely, we believe) that the three-way junction and hairpin structures are both formed in infected cells, possibly a consequence of heterogeneous transcription start site usage (see below) [42].

## 4. Probing the Intermolecular Interface in the Dimeric 5′-Leader by NMR

**Figure 3:**
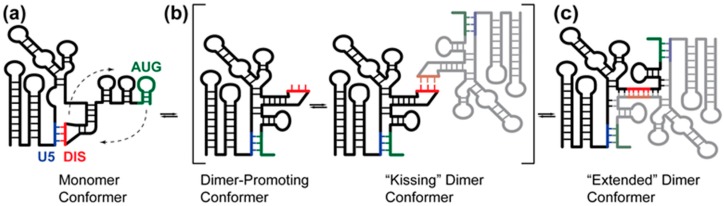
Proposed mechanism of HIV-1 5′-leader dimerization. (**a**) The palindromic DIS loop is sequestered in base pairing with the U5 region in the monomer conformation; (**b**) AUG can compete for base pairing with U5, displacing and exposing the DIS. The exposure of DIS enables the formation on a “kissing” dimer, where two RNA molecules interact at the palindromic DIS loop sequence. There is no spectroscopic evidence for this conformation; therefore, it is likely a short-lived species; (**c**) the HIV-1 5′-leader rapidly adopts an “extended” dimer conformation characterized by extensive base pairing between the two RNA molecules. (Figure adapted from [31]).

The 5′-leader contains a conserved stem-loop that serves as the primary site of genome DIS [15,43,44,45,46,47,48,49,50]. The isolated DIS oligoribonucleotide is capable of adopting either a “kissing” dimer, where the only intermolecular contacts are in the palindromic loop (5′-GCGCGC-3′ for HIV-1_NL4-3_) [51,52,53,54,55], or an “extended” dimer with extensive intermolecular interactions [54,56,57]. Longer DIS oligoribonucleotides which contain unpaired or bulged residues are able to convert from “kissing” to thermodynamically stable “extended” dimers [58]. Native gel electrophoresis studies indicate that 5′-leader RNAs of some lentiviruses, including some strains of HIV-1, form “labile” dimers [34,59,60] presumed to be mediated by loop–loop “kissing” interactions, and that the labile dimers can in some cases be converted by mild heating to “non-labile” dimers presumed to be stabilized by an extended DIS duplex interface [61]. In contrast, only non-labile dimers have been observed for the HIV-1_NL4-3_ 5′-leader. The propensity to form observable labile dimers appears to be related to the GC content of the DIS palindrome, since labile dimers were only observed for leaders containing a GCGCGC DIS sequence [34]. Using a ^2^H-edited NMR spectroscopy approach, the dimer interface of the HIV-1_NL4-3_ 5′-leader was shown to include both extended DIS duplex interactions and intermolecular U5:AUG base pairing, Figure 3 [31]. Using a differential mutagenesis/^2^H-edited 1D NMR approach, the U5:AUG intermolecular interface was shown to form on roughly the same timescale as overall dimerization, suggesting that the proposed kissing intermediate, if formed, converts rapidly to the extended interface structure at 35 °C even in the absence of RNA chaperones [31].

## 5. Transcriptional Start Site Heterogeneity Modulates Genome Structure and Function

An additional layer of regulation may come from the sequence of the RNA itself, determined by the transcription start site (TSS) used during genome synthesis [42,62]. Shortly after initiation of RNA synthesis, the HIV-1 genome is co-transcriptionally capped by a 5′-5′ triphosphate linked 7-methylguanosine moiety (7MeG) [62,63,64,65,66]. The U3/R junction of HIV-1 proviruses contains a conserved run of three consecutive guanosines, any (or all) of which can serve as the +1 site for RNA transcription. Recent reports suggest that HIV-1 utilizes three different transcription start sites (1G, 2G, and 3G), creating an array of genomes with varying 5′ end identity, each of which is co-transcriptionally capped (^Cap^1G, ^Cap^2G, ^Cap^3G). Interestingly, the ^Cap^1G genome is predominately packaged into virions (70 to ~100%) [42,62], whereas ^Cap^2G and ^Cap^3G RNAs are enriched on polysomes [42]. The distinct function of these transcripts appears to correlate with a distinct structural rearrangement. In vitro transcribed 5′-leader (5′-L) RNAs beginning with either 1G, 2Gs, or 3Gs displayed strikingly different dimerization profiles, with 1G and 2G 5′-L RNA favoring the dimer and 3G 5′-L RNA favoring the monomer [42]. The authors went on to examine the effect that capping played on these in vitro transcribed leader RNAs. Dimerization propensities of ^Cap^1G, ^Cap^2G, ^Cap^3G 5′-L RNAs were assayed and, strikingly, the influence of a 7MeG cap mimicked that of the addition of a single guanosine (^Cap^1G 5′-L and 2G 5′-L behaved similarly). Mutagenesis studies show that destabilizing the predicted base of the PolyA stem promoted the monomeric RNA conformation while stabilizing the base of the hairpin promoted dimerization. These studies suggest a new paradigm for RNA fate, in which function is not encoded by an intrinsic monomer–dimer equilibrium, but rather by the transcriptionally encoded sequence of the capped 5′-terminus [42].

## 6. Conclusions

### 6.1. Strengths and Limitations of NMR Spectroscopy for Large RNAs

Although NMR spectroscopy has historically been used to determine structures of relatively small RNAs that typically comprise fewer than 60 nucleotides [67,68,69,70,71,72,73,74,75], the above studies have shown that structures can be probed by NMR spectroscopy in RNAs comprising up to 688 nucleotides. When used in combination with cryo-electron microscopy (cryoEM) or small-angle X-ray scattering (SAXS), 3D structural information can be obtained for relatively large protein:RNA complexes [68,69]. An advantage of the Adenosine-H2 detected, ^2^H-edited method described above is that it enables direct detection of structure, even in RNAs that exist as equilibrium mixtures of species. The fact that HIV-1 5′-leader transcripts can exist as an equilibrium mixture of conformers in vitro, and as an equilibrium mixture of chemical species and structures in transfected cells, may explain discrepant secondary structure predictions made on the basis of chemical probing [37,38,39,76], and it is noteworthy that the NMR-derived structure of the HIV-1 packaging signal [36] is more consistent with in-gel Selective 2′-Hydroxyl Acylation analyzed by Primer Extension (SHAPE) probing of the dimeric 5′-leader [37] than previously published SHAPE-derived models [38,39]. Difficulties interpreting bulk chemical probing data may now be overcome using a “mutate-and-map” approach coupled with the RNA Ensemble Extraction From Footprinting Instights Tool (REEFFIT) algorithm in order to deconvolute and reconstruct complex conformational landscapes [77].

Disadvantages of the NMR spectroscopy approach are the time and expense associated with data collection (up to ~five days per spectrum for the largest RNAs) and sample preparation (up to a dozen differentially ^2^H-labeled RNAs at ~$1000 per sample). One key weakness of the NMR spectroscopy approach described above is that it only involved the use of NOE-derived structural information, which is inherently short-range (~5 Å, although some very long-range NOEs were identified in highly deuterated samples [36]). For appropriate RNAs that adopt a unique structure and do not undergo conformational averaging, NMR-derived residual dipolar couplings (RDCs) can provide global structural information regarding the relative orientations of the different helices [78,79]. The use of hybrid NMR/SAXS [78,79] or NMR/cryoEM [80] approaches provides exciting new opportunities to combine the high resolution “local” structural information obtainable by NMR spectroscopy with lower resolution global structural information derived by SAXS and/or cryoEM. Of course, caution must be taken when using RDC [80], cryoEM, or SAXS data to refine structures of RNAs that undergo rapid, large-scale conformational averaging. In addition, although the size limit for RNA NMR spectroscopy studies remains undefined, it will certainly not approach the large molecular sizes that are now tractable by X-ray crystallography and recently developed high resolution (~3 Å) cryoEM methods [81].

### 6.2. Future Directions

Recent advances in isotopic labeling, segmental ligation, and non-covalent fragmentation-based labeling approaches have expanded the size of RNAs that can be successfully studied by NMR spectroscopy. Due in large part to these technological advances, high resolution structures have been obtained for relatively large, independently functional portions of the HIV-1 5′-leader, and secondary structures have been directly detected in the intact leader [13,31,36]. However, these studies still do not provide a complete view of all functional elements within the dimeric form of the HIV-1 5′- leader, and the monomeric form of the leader has not been extensively probed by NMR (shaded regions, Figure 1a). A number of important questions remain: What is the structure of the intact 5′-leader in both its monomeric and dimeric states? How does the structure of the genomic 5′-leader compare to that of various spliced viral mRNAs? How does transcription start site usage influence RNA structure and function? Ultimately, it will be important to determine if these new NMR approaches can contribute to our understanding of the Gag:Ψ^CES^ structures that assemble in cells and nucleate virus assembly. Although NMR alone will not likely be capable of providing all of the information needed to determine 3D structures of large Gag:5′-leader complexes, it could provide important complementary information when used in combination with other techniques such as cryo-EM [80] or SAXS [69,82].

## References

[1] Coffin J.M., Hughes S.H., Varmus H.E. (1997). Retroviruses.

[2] Hu W.-S., Temin H.M. (1990). Genetic consequences of packaging two RNA genomes in one retroviral particle: Pseudodiploidy and high rate of genetic recombination. Proc. Natl. Acad. Sci. USA.

[3] Hu W.S., Temin H.M. (1990). Retroviral recombination and reverse transcription. Science.

[4] Onafuwa-Nuga A., Telesnitsky A. (2009). The remarkable frequency of human immunodeficiency virus type 1 genetic recombination. Microbiol. Mol. Biol. Rev..

[5] Nora T., Charpentier C., Tenaillon O., Hoede C., Clavel F., Hance A.J. (2007). Contribution of recombination to the evolution of human immunodeficiency viruses expressing resistance to antiretroviral treatment. J. Virol..

[6] Schwartz S., Felber B.K., Benko D.M., Fenyo E.M., Pavlakis G.N. (1990). Cloning and functional analysis of multiply spliced mRNA species of human immunodeficiency virus type 1. J. Virol..

[7] Nikolaitchik O.A., Dilley K.A., Fu W., Gorelick R.J., Tai S.H., Soheilian F., Ptak R.G., Nagashima K., Pathak V.K., Hu W.-S. (2013). Dimeric RNA recognition regulates HIV-1 genome packaging. PLoS Pathog..

[8] Lu K., Heng X., Summers M.F. (2011). Structural determinants and mechanism of HIV-1 genome packaging. J. Mol. Biol..

[9] Lever A.M. (2007). HIV-1 RNA packaging. Adv. Pharmacol..

[10] Paillart J.-C., Shehu-Xhilaga M., Marquet R., Mak J. (2004). Dimerization of retroviral RNA genomes: An inseparable pair. Nat. Rev. Microbiol..

[11] Greatorex J. (2004). The retroviral RNA dimer linkage: Different structures may reflect different roles. Retrovirology.

[12] Abbink T.E.M., Berkhout B. (2008). RNA structure modulates splicing efficiency at the human Immunodeficiency Virus Type 1 major splice donor. J. Virol..

[13] Lu K., Heng X., Garyu L., Monti S., Garcia E., Kharytonchyk S., Dorjsuren B., Kulandaivel G., Jones S., Hiremath A. (2011). NMR detection of structures in the HIV-1 5′-leader RNA that regulate genome packaging. Science.

[14] Kuzembayeva M., Dilley K., Sardo L., Hu W.-S. (2014). Life of PSI: How full-length HIV-1 RNAs become packaged genomes in the viral particles. Virology.

[15] Moore M.D., Fu W., Nikolaitchik O., Chen J., Ptak R.G., Hu W.-S. (2007). Dimer initiation signal of human immunodeficiency virus type 1: Its role in partner selection during RNA copackaging and its effects on recombination. J. Virol..

[16] Moore M.D., Nikolaitchik O.A., Chen J., Hammarskjold M.L., Rekosh D., Hu W.S. (2009). Probing the HIV-1 genomic RNA trafficking pathway and dimerization by genetic recombination and single virion analyses. PLoS Pathog..

[17] Russell R.S., Hu J., Laughrea M., Wainberg M.A., Liang C. (2002). Deficient dimerization of human immunodeficiency virus type 1 RNA caused by mutations of the U5 RNA sequences. Virology.

[18] Nikolaitchik O., Rhodes T.D., Ott D., Hu W.-S. (2006). Effects of mutations in the Human Immunodeficiency Virus Type 1 gag gene on RNA packaging and recombination. J. Virol..

[19] Song R., Kafaie J., Laughrea M. (2008). Role of the 5’ TAR stem--loop and the U5-AUG duplex in dimerization of HIV-1 genomic RNA. Biochemistry.

[20] Jouvenet N., Simon S.M., Bieniasz P.D. (2009). Imaging the interaction of HIV-1 genomes and Gag during assembly of individual viral particles. Proc. Natl. Acad. Sci. USA.

[21] Berkowitz R., Fisher J., Goff S.P. (1996). RNA packaging. Curr. Top. Microbiol. Immunol..

[22] D’Souza V., Summers M.F. (2005). How retroviruses select their genomes. Nat. Rev. Microbiol..

[23] Greatorex J., Lever A. (1998). Retroviral RNA dimer linkage. J. Gen. Virol..

[24] Hellmund C., Lever A.M. (2016). Coordination of Genomic RNA Packaging with Viral Assembly in HIV-1. Viruses.

[25] Jewell N.A., Mansky L.M. (2000). In the beginning: Genome recognition, RNA encapsidation and the initiation of complex retrovirus assembly. J. Gen. Virol..

[26] Mailler E., Bernacchi S., Marquet R., Paillart J.C., Vivet-Boudou V., Smyth R.P. (2016). The Life-Cycle of the HIV-1 Gag-RNA Complex. Viruses.

[27] Paillart J.-C., Marquet R., Skripkin E., Ehresmann C., Ehresmann B. (1996). Dimerization of retroviral genomic RNAs: Structural and functional implications. Biochimie.

[28] Rein A. (1994). Retroviral RNA packaging: A review. Arch. Virol..

[29] Russell R.S., Liang C., Wainberg M.A. (2004). Is HIV-1 RNA dimerization a prerequisite for packaging? Yes,no, probably?. Retrovirology.

[30] Ganser-Pomillos B.K., Yeager M., Sundquist W.I. (2008). The structural biology of HIV assembly. Curr. Opin. Struct. Biol..

[31] Keane S.C., Van V., Frank H.M., Sciandra C.A., McCowin S., Santos J., Heng X., Summers M.F. (2016). NMR detection of intermolecular interaction sites in the dimeric 5’-leader of the HIV-1 genome. Proc. Natl. Acad. Sci. USA.

[32] Abbink T.E.M., Berkhout B. (2003). A novel long distance base-pairing interaction in Human Immunodeficiency Virus Type 1 RNA occludes the Gag start codon. J. Biol. Chem..

[33] Damgaard C.K., Andersen E.S., Knudsen B., Gorodkin J., Kjems J. (2004). RNA interactions in the 5’ region of the HIV-1 genome. J. Mol. Biol..

[34] Tran T., Liu Y., Marchant J., Monti S., Seu M., Zaki J., Yang A.L., Bohn J., Ramakrishnan V., Singh R. (2015). Conserved determinants of lentiviral genome dimerization. Retrovirology.

[35] Heng X., Kharytonchyk S., Garcia E.L., Lu K., Sachin Divakaruni S., LaCotti C., Edme K., Telesnitsky A., Summers M.F. (2012). Identification of a minimal HIV-1 RNA packaging signal. J. Mol. Biol..

[36] Keane S.C., Heng X., Lu K., Kharytonchyk S., Ramakrishnan V., Carter G., Barton S., Hosic A., Florwick A., Santos J. (2015). Structure of the HIV-1 RNA packaging signal. Science.

[37] Kenyon J.C., Prestwood L.J., Le Grice S.F., Lever A.M. (2013). In-gel probing of individual RNA conformers within a mixed population reveals a dimerization structural switch in the HIV-1 leader. Nucleic Acids Res..

[38] Wilkinson K.A., Gorelick R.J., Vasa S.M., Guex N., Rein A., Mathews D.H., Giddings M.C., Weeks K.M. (2008). High-throughput SHAPE analysis reveals structures in HIV-1 genomic RNA strongly conserved across distinct biological states. PLoS Biol..

[39] Watts J.M., Dang K.K., Gorelick R.J., Leonard C.W., Bess J.W., Swanstrom R., Burch C.L., Weeks K.M. (2009). Architecture and secondary structure of an entire HIV-1 RNA genome. Nature.

[40] Dirac A.M.G., Huthoff H., Kjems J., Berkhout B. (2001). The dimer initiation site hairpin mediates dimerization of the human immunodeficiency virus, type 2 RNA genome. J. Biol. Chem..

[41] Smyth R.P., Despons L., Huili G., Bernacchi S., Hijnen M., Mak J., Jossinet F., Weixi L., Paillart J.C., von Kleist M. (2015). Mutational interference mapping experiment (MIME) for studying RNA structure and function. Nat. Methods.

[42] Kharytonchyk S., Monti S., Smaldino P.J., Van V., Bolden N.C., Brown J.D., Russo E., Swanson C., Shuey A., Telesnitsky A. (2016). Transcriptional start site heterogeneity modulates the structure and function of the HIV-1 genome. Proc. Natl. Acad. Sci. USA.

[43] Kim H.-J., Lee K., O’Rear J.J. (1994). A short sequence upstream of the 5’ major splice site is important for encapsidation of HIV-1 genomic RNA. Virology.

[44] Skripkin E., Paillart J.C., Marquet R., Ehresmann B., Ehresmann C. (1994). Identification of the primary site of the human immunodefieiency virus type 1 RNA dimerization in vitro. Proc. Natl. Acad. Sci. USA.

[45] Berkhout B., Vastenhouw N.L., Klasens B.I., Huthoff H. (2001). Structural features in the HIV-1 repeat region facilitate strand transfer during reverse transcription. RNA.

[46] Clever J.L., Wong M.L., Parslow T.G. (1996). Requirements for kissing-loop-mediated dimerization of human immunodeficiency virus RNA. J. Virol..

[47] Paillart J.-C., Skripkin E., Ehresmann B., Ehresmann C., Marquet R. (1996). A loop–loop “kissing” complex is the essential part of the dimer linkage of genomic HIV-1 RNA. Proc. Natl. Acad. Sci. USA.

[48] Paillart J.C., Berthoux L., Ottmann M., Darlix J.L., Marquet R., Ehresmann B., Ehresmann C. (1996). A dual role of the putative RNA dimerization initiation site of human immunodeficiency virus type 1 in genomic RNA packaging and proviral DNA synthesis. J. Virol..

[49] McBride M.S., Panganiban A.T. (1996). The human immunodeficiency virus type 1 encapsidation site is a multipartite RNA element composed of functional hairpin structures. J. Virol..

[50] Berkhout B., van Wamel J.L. (1996). Role of the DIS hairpin in replication of human immunodeficiency virus type 1. J. Virol..

[51] Mujeeb A., Clever J.L., Billeci T.M., James T.L., Parslow T.G. (1998). Structure of the dimer initiation complex of HIV-1 genomic RNA. Nat. Struct. Biol..

[52] Dardel F., Marquet R., Ehresmann C., Ehresmann B., Blanquet S. (1998). Solution studies of the dimerization initiation site of HIV-1 genomic RNA. Nucleic Acids Res..

[53] Ennifar E., Walter P., Ehresmann B., Ehresmann C., Dumas P. (2001). Crystal structures of coaxially stacked kissing complexes of the HIV-1 RNA dimerization initiation site. Nat. Struct. Biol..

[54] Baba S., Takahashi K., Noguchi S., Takaku H., Koyanagi Y., Yamamoto N., Kawai G. (2005). Solution RNA structures of the HIV-1 dimerization initiation site in the kissing-loop and extended-duplex dimers. J. Biochem..

[55] Kieken F., Paquet F., Brule F., Paoletti J., Lancelot G. (2006). A new NMR solution structure of the SL1 HIV-1Lai loop–loop dimer. Nucleic Acids Res..

[56] Mujeeb A., Parslow T.G., Zarrinpar A., Das C., James T.L. (1999). NMR structure of the mature dimer initiation complex of HIV-1 genomic RNA. FEBS Lett..

[57] Ulyanov N.B., Mujeeb A., Du Z., Tonelli M., Parslow T.G., James T.L. (2006). NMR structure of the full-length linear dimer of stem-loop-1 RNA in the HIV-1 dimer initiation site. J. Biol. Chem..

[58] Takahashi K.-I., Baba S., Chattopadhyay P., Koyanagi Y., Yamamoto N., Takaku H., Kawai G. (2000). Structural requirement for the two-step dimerization of human immunodeficiency virus type-1 genome. RNA.

[59] Laughrea M., Jette L. (1997). HIV-1 genome dimerization: Kissing-loop hairpin dictates whether nucleotides downstream of the 5’ splice junction contribute to loose and tight dimerization of human immunodeficiency virus RNA. Biochemistry.

[60] Marquet R., Paillart J.-C., Skripkin E., Ehresmann C., Ehresmann B. (1994). Dimerization of human immunodeficiency virus type 1 RNA involves sequences located upstream of the splice donor site. Nucl. Acids Res..

[61] Laughrea M., Jette L. (1996). Kissing-loop model of HIV-1 genome dimerization: HIV-1 RNAs can assume alternative dimeric forms, and all sequences upstream or downstream of Hairpin 248-271 are dispensable for dimer formation. Biochemistry.

[62] Masuda T., Sato Y., Huang Y.L., Koi S., Takahata T., Hasegawa A., Kawai G., Kannagi M. (2015). Fate of HIV-1 cDNA intermediates during reverse transcription is dictated by transcription initiation site of virus genomic RNA. Sci. Rep..

[63] Chiu Y.L., Coronel E., Ho C.K., Shuman S., Rana T.M. (2001). HIV-1 Tat protein interacts with mammalian capping enzyme and stimulates capping of TAR RNA. J. Biol. Chem..

[64] Zhou M., Deng L., Kashanchi F., Brady J.N., Shatkin A.J., Kumar A. (2003). The Tat/TAR-dependent phosphorylation of RNA polymerase II C-terminal domain stimulates cotranscriptional capping of HIV-1 mRNA. Proc. Natl. Acad. Sci. USA.

[65] Menees T.M., Muller B., Krausslich H.G. (2007). The major 5’ end of HIV type 1 RNA corresponds to G456. AIDS Res. Hum. Retrovir..

[66] Sharma A., Yilmaz A., Marsh K., Cochrane A., Boris-Lawrie K. (2012). Thriving under stress: Selective translation of HIV-1 structural protein mRNA during Vpr-mediated impairment of eIF4E translation activity. PLoS Pathog..

[67] Wang Y., Yesselman J.D., Zhang Q., Kang M., Feigon J. (2016). Structural conservation in the template/pseudoknot domain of vertebrate telomerase RNA from teleost fish to human. Proc. Natl. Acad. Sci. USA.

[68] Jiang J., Chan H., Cash D.D., Miracco E.J., Ogorzalek Loo R.R., Upton H.E., Cascio D., O’Brien Johnson R., Collins K., Loo J.A., Zhou Z.H., Feigon J. (2015). Structure of Tetrahymena telomerase reveals previously unknown subunits, functions, and interactions. Science.

[69] Jain N., Morgan C.E., Rife B.D., Salemi M., Tolbert B.S. (2016). Solution Structure of the HIV-1 Intron Splicing Silencer and Its Interactions with the UP1 Domain of Heterogeneous Nuclear Ribonucleoprotein (hnRNP) A1. J. Biol. Chem..

[70] Kang M., Eichhorn C.D., Feigon J. (2014). Structural determinants for ligand capture by a class II preQ1 riboswitch. Proc. Natl. Acad. Sci. USA.

[71] Kruschel D., Skilandat M., Sigel R.K. (2014). NMR structure of the 5’ splice site in the group IIB intron Sc.ai5gamma--conformational requirements for exon-intron recognition. RNA.

[72] Popovic M., Greenbaum N.L. (2014). Role of helical constraints of the EBS1-IBS1 duplex of a group II intron on demarcation of the 5’ splice site. RNA.

[73] Cornish P.V., Hennig M., Giedroc D.P. (2005). A loop 2 cytidine-stem 1 minor groove interaction as a positive determinant for pseudoknot-stimulated -1 ribosomal frameshifting. Proc. Natl. Acad. Sci. USA.

[74] Lawrence D.C., Stover C.C., Noznitsky J., Wu Z.-R., Summers M.F. (2003). Structure of the intact stem and bulge of HIV-1 ψ-RNA stem loop SL1. J. Mol. Biol..

[75] Amarasinghe G.K., De Guzman R.N., Turner R.B., Chancellor K., Wu Z.-R., Summers M.F. (2000). NMR structure of the HIV-1 nucleocapsid protein bound to stem-loop SL2 of the Y-RNA packaging signal. J. Mol. Biol..

[76] Deforges J., Chamond N., Sargueil B. (2012). Structural investigation of HIV-1 genomic RNA dimerization process reveals a role for the Major Splice-site Donor stem loop. Biochimie.

[77] Cordero P., Das R. (2015). Rich RNA Structure Landscapes Revealed by Mutate-and-Map Analysis. PLoS Comput. Biol..

[78] Zuo X., Wang J., Foster T.R., Schwieters C.D., Tiede D.M., Butcher S.E., Wang Y.-X. (2008). Global molecular structure and interfaces: Refining an RNA:RNA complex structure using solution X-ray scattering data. J. Am. Chem. Soc..

[79] Wang J., Zuo X., Yu P., Xu H., Starich M.R., Tiede D.M., Shapiro B.A., Schwieters C.D., Wang Y.X. (2009). A method for helical RNA global structure determination in solution using small-angle x-ray scattering and NMR measurements. J. Mol. Biol..

[80] Miyazaki Y., Irobalieva R.N., Tolbert B.S., Smalls-Manty A., Iyalla K., Loeliger K., D’Souza V., Khant H., Schmid M.F., Garcia E. (2010). Structure of a conserved retroviral RNA packaging element by NMR spectroscopy and cryo-electron tomography. J. Mol. Biol..

[81] Fischer N., Neumann P., Konevega A.L., Bock L.V., Ficner R., Rodnina M.V., Stark H. (2015). Structure of the *E. coli* ribosome-EF-Tu complex at <3 A resolution by Cs-corrected cryo-EM. Nature.

[82] Fang X., Wang J., O’Carroll I.P., Mitchell M., Zuo X., Wang Y., Yu P., Liu Y., Rausch J.W., Dyba M.A. (2013). An unusual topological structure of the HIV-1 Rev response element. Cell.

